# Trace Element Contents in Maize following the Application of Organic Materials to Reduce the Potential Adverse Effects of Nitrogen

**DOI:** 10.3390/ma16010215

**Published:** 2022-12-26

**Authors:** Mirosław Wyszkowski, Marzena S. Brodowska, Natalia Kordala

**Affiliations:** 1 Department of Agricultural and Environmental Chemistry, University of Warmia and Mazury, Łódzki 4 Sq., 10-727 Olsztyn, Poland; 2 Department of Agricultural and Environmental Chemistry, University of Life Sciences, Akademicka 15 Str., 20-950 Lublin, Poland

**Keywords:** organic materials, nitrogen, soils, maize, trace elements

## Abstract

The plants cultivated in loamy sand contained less iron, manganese, copper, cobalt, nickel, and zinc while containing more chromium, lead, and cadmium than in sand. This study was launched to use organic materials in the form of humic acids (HA) to reduce the potential negative effects of excessive nitrogen fertiliser (ammonium nitrate, urea, and urea and ammonium nitrate solution—UAN) application rates (160 mg N kg^−1^ soil) on the trace element contents in maize in two soils differing in granulometric composition. HA were applied into the soil three times during the maize vegetation: before the sowing, at the five-leaf unfolded stage, and at the intensive shoot growth stage. The HA doses amounted to 0, 0.05, 0.10, and 0.15 g kg^−1^ soil. Urea fertilisation increased the cadmium, lead, chromium, and nickel contents and reduced the iron content in maize on both soils. UAN contributed to an increased chromium content being higher than that caused by urea and to reduced iron content in the aboveground parts of maize, as compared to the objects with ammonium nitrate. In the series with ammonium nitrate, the highest dose of HA reduced the manganese, zinc, iron, and cobalt contents in maize on both soils. In the series with urea, however, their reducing effect on the copper and iron contents in maize on both soils was noted. The study also demonstrated a positive reduction in the contents of many other trace elements in maize under the influence of the application of HA (particularly, their highest dose). However, it only concerned one of the soils under study. The application of HA into the soil can be effective in reducing the trace element content in plants and can mitigate the adverse environmental impact of intensive agricultural production.

## 1. Introduction

In this era of massive intensification of crop production, maintaining soils in a state of adequate fertility is among the basic tasks of crop production techniques. The fundamental crop production treatment that affects plant productivity and yield quality and regulates the soil agrochemical properties is fertilisation [1,2]. 

Rationalisation of the use of mineral and organic fertilisers contributes to the sustainable management of plant nutrients and soil organic matter [3]. Of all the basic nutrients, nitrogen is one of the most yield-forming [4]. However, intensive agricultural practices are raising specific concerns about the possible occurrence of adverse effects from applying very high fertiliser doses [5]. The excessive and inappropriate use of nitrogen fertilisers reduces soil fertility by upsetting the ionic balance between components, reducing microbial growth and activity and inducing salinity [6]. Over-fertilisation can also contribute to groundwater and surface water contamination through nitrate leaching [7], exacerbate the so-called “greenhouse effect” and the acid rain phenomenon [8], and induce acidification. Under reduced pH conditions, the amount of readily soluble forms of trace elements in the soil increases, as does their uptake and accumulation by plants [9]. Therefore, indirectly, nitrogen fertilisation can increase the availability of trace elements, which become hazardous in greater amounts. What is more, the use of mineral nitrogen fertilisers (particularly urea) accounts for 19.0–20.3% of the total atmospheric ammonia emissions [5,10]. This gas exerts a harmful effect on the environment and human health. Its dry and wet deposition results in soil acidification, eutrophication of natural aquatic ecosystems, and reduced biodiversity of the biosphere [10]. Ammonia is also a secondary source of NO_2_ and CO_2_, thus leading to ozone depletion in the stratosphere [10]. For this reason, measures need to be taken to help increase the efficiency of fertilisation management, which would allow sustainable agricultural development to be maintained and environmentally friendly agriculture to be achieved [11]. 

Maize (*Zea mays* L.), due to its large cultivation area and its importance in food and fodder economy, is a plant often used in scientific research. Maize is one of the most widely grown crops worldwide [12]. Its cultivation, however, is exhausting [13] and requires intensive nitrogen fertilisation. Maize’s need for nitrogen is determined by the stage of development: at the beginning of growing, they are minimal, while they reach a peak during the flowering and at the beginning of grain formation [14,15]. The recovery of applied nitrogen by maize rarely exceeds 50% [15,16]. The environmental cost of the excessive application of nitrogen fertilisers in Europe is estimated at USD 78–357 billion per annum [15,17]. Therefore, the optimal supply of nitrogen fertilisers is one of the basic principles of sustainable agricultural development that is aimed at increasing the total biomass production and the crop yield while ensuring the environmental safety of the soil [5,18].

The adverse effect of excessive doses of nitrogen fertilizers can be reduced by applying various organic substances [19,20,21] to the soil, such as humic acids (HA), biochar, or mineral additives [22], such as zeolites or bentonites, and others. The use of organic fertilisers increases the amount of humus in the soil, which is essential for proper plant nutrition [19]. The role served by organic substances in the soil-forming processes, and the development of soil properties is enormous. The amount and quality of humic compounds regulate the reduction–oxidation and thermal relationships and determine the buffering and sorption properties in biologically active soils [23]. The basic components of organic matter in the soil include macromolecular, amorphous humic substances (up to 85%), with the remaining proportion comprising the plant roots (10%) and microorganisms (5%) [24]. Humic substances can be divided into three fractions according to their solubility [25]. These are, respectively: fulvic acids (FA, soluble at all pH values), humic acids (soluble in the alkaline environment, and insoluble at pH of 1.0), and humins (HN, insoluble at all pH values) [24].

Humic substances have a positive effect on soil fertility [26] and improve its physical and biochemical properties, mainly due to the high cation exchange sorption and high water-holding capacity [27,28]. Additionally, they improve soil structure and texture and increase the population of beneficial microorganisms [27,29,30]. By binding with clay minerals and iron oxides, they provide soil aggregates with stability and water resistance, thus creating better soil permeability and offering optimum air and water conditions for plants [28]. Humic substances also contribute to the improved uptake of macro- and microelements by plants thanks to the chelation and co-transportation of these components to plants [31,32]. They mainly increase the phytoavailability of phosphorus, potassium, magnesium, iron, and zinc [33,34]. Numerous studies have confirmed the beneficial effects of HA and FA on plant yielding [35], nutrient metabolism, and the synthesis of compounds involved in plant adaptation to stress factors [28,33,36]. What is more, humic substances promote seed germination and seedling development [37,38] and root growth [39,40] and increase the cell membrane permeability and stimulate the synthesis of nucleotides [41], chlorophyll [42], proteins, and monosaccharides [35]. Thanks to its ability to bind and precipitate harmful substances, soil humus also serves a protective function in the environment [43] and affects the behaviour and availability of trace elements in the soil [44]. The HA fraction contains approx. 60% organic carbon [28], which makes it a significant link in the global cycle of this element and an additional carbon source for soil microorganisms responsible for the biological activity of the soil [27]. Scientific experiments have successfully demonstrated a positive relationship between the use of HA and the enzymatic processes in the soil profile [30], especially in terms of acid and alkaline phosphatase activity and urease inhibition [45].

The application of HA to the soil can reduce the adverse effects of intensive agricultural production and the excessive use of mineral fertilisers, particularly nitrogen fertilisers [46]. Humic substances increase the efficiency of these fertilisers and prolong their action [47] and increase the nitrogen use efficiency (NUE) [42]. Due to their polymeric structure and large specific surface area, the above-mentioned substances are involved in non-ionic forms of elemental binding in soils, which makes it possible for their use to also affect the content of bioavailable trace element fractions [48]. These elements are not biodegradable and, therefore, constitute a persistent environmental pollutant [49]. Their toxic levels in the soil can adversely affect plant growth and reduce yields [50], as well as pose a hazard to human health due to bioaccumulation in the food chain [51].

The sustainable management of fertiliser components in agriculture is of economic as well as environmental significance. It is, therefore, necessary to search for an effective fertilisation strategy that simultaneously enables: (1) the maintenance of soil productivity, (2) the realisation of the crop potential, and (3) the reduction in the environmental impact [52].

In view of the above, we hypothesized that the application of HA to the soil would limit the potential negative effect of nitrogen fertilisation on the content of trace elements in maize (*Zea mays* L.). This led to detailed predictions that: (1) nitrogen fertilisation in the form of ammonium nitrate, urea, and UAN would increase the content of trace elements in the aerial parts of maize, (2) the content of trace elements in maize would higher on sand than loamy sand, and (3) the application of HA to the soil would limit the effect of nitrogen fertilisation on the contents of trace elements in plants.

## 2. Materials and Methods

### 2.1. Methodology of the Vegetation Experiment

The vegetation pot experiment was conducted under the three-factor experimental design. The first-order factor was the application of organic material in the form of HA, while the second-order factor included different types of nitrogen fertilisers, and the third-order factor comprised two soils characterised by a different granulometric composition. HA (Calfert, Warsaw, Poland) were applied into the soil three times during the maize vegetation: before the sowing, at the five-leaf unfolded stage, and at the intensive shoot growth stage. Their total doses amounted to 0, 0.05, 0.10, and 0.15 g kg^−^^1^ soil. The nitrogen dose (Grupa Azoty S.A., Tarnów, Poland) incorporated pre-sowing into the soil in the form of ammonium nitrate (34% N), urea (46% N), and the urea and ammonium nitrate solution, UAN (32% N), was identical on all objects (160 mg N kg^−^^1^ soil). The study was conducted on two soils gathering from 0-25 cm layer differing in their granulometric compositions, i.e., sand (>0.05 mm sand—91.88%, 0.002–0.05 mm silt—7.44%, and <0.002 mm clay—0.68%) and loamy sand (>0.05 mm sand—77.55%, 0.002–0.05 mm silt—19.95%, and <0.002 mm clay—2.50%) [53]. The soils were taken from the humus layer. Before setting up an experiment, the soils were sieved through a sieve with a diameter of 1 cm. Phosphorus and potassium were also applied into each pot at an amount of 60 mg P (Super FosDar 40, Grupa Azoty „Fosfory” Sp. z o.o., Gdańsk, Poland); 170 mg K per kg soil (KCl). Detailed properties of the soil and the chemical composition of HA are provided in a previously published paper [46]. 

The soil, in the amount of 9 kg, was thoroughly mixed with mineral (nitrogen, phosphate, and potassium) fertilisers and a starting dose of HA and placed into polyethylene pots. The test plant was maize (*Zea mays* L.) of the Kadryl Polish cultivar (Małopolska Hodowla Roślin Sp. zo.o., Kraków, Poland). The plant density was six plants per pot. During the plant growing season, the moisture was maintained at a constant level of 60% of the maximum water capacity. The leaf greenness index SPAD was measured in the stages of the fifth leaf unfolded, intensive stem elongation, and tasselling of maize. Detailed results of the SPAD index during the vegetative growth of maize were earlier published [54]. During the maize harvest, at the end of the heading (inflorescence fully emerged stage, BBCH 59), samples of plant material were collected for laboratory analyses.

### 2.2. Methodology of Laboratory Testing and Statistical Analyses

The plant material samples, following crushing, drying at 60 °C in a Binder FED720 drying and heating chamber (Binder GmbH, Tuttlingen, Germany), and grinding using the cutting mill SM 200 (Retsch GmbH, Haan, Germany), were wet-digested in concentrated 65% nitric acid (HNO_3_, analytical grade, with a density of 1.40 g cm^−^^3^). The mineralisation was carried out in the Xpress Teflon® vessels in a MARS 6 microwave digestion system (CEM Corporation, Matthews, NC, USA), according to the methodology US-EPA3051 [54]. The determination of the trace element contents (Cd, Pb, Cr, Ni, Zn, Cu, Mn, Fe, and Co) was carried out by the atomic absorption spectroscopy (AAS) method using a SpectrAA 240FS spectrophotometer (Varian Inc., Mulgrave, Australia) [55]. For the purposes of quality control of the analyses of trace element contents, standard materials of the company Fluka (Charlotte, NC, USA, Cd 51994, Pb 16595, Cr 02733, Ni 42242, Zn 188227, Cu 38996, Mn 63534, Fe 16596, and Co 119785.0100), and the Certified Analytical Reference Material NCS ZC 73,030 (Chinese National Analysis Centre for Iron and Steel, Beijing, China) were used. The chlorophyll meter Minolta SPAD-502Plus (Konica Minolta Sensing Europe B.V., Nieuwegein, the Netherlands) was used for measurements of the SPAD leaf greenness index [56]. The Mastersizer 3000 (Malvern Instruments Ltd., Worcestershire, UK) was used for laser measurements of the soil texture (granulometric) composition [57].

The Shapiro–Wilk tests were used to verify of the distribution normality of the data. The statistical processing of the study results used the Statistica program [58] and applied the three-factor analysis of variance ANOVA [58], HSD Tukey’s test (*p* ≤ 0.01) [58], principal component analysis (PCA) [54] using multidimensional explorative techniques, and the percentage of the observed variation calculated with the η2 coefficient and the ANOVA method [58]. Homogeneous groups were identified at a significance level of *p* ≤ 0.01. PCA was used to illustrate the relationship (correlation) between contents of trace elements (Cd, Pb, Cr, Ni, Zn, Cu, Mn, Fe, and Co) in maize and the SPAD index in stages of the 5th leaf unfolded, stem elongation, and tasselling; height; aerial parts fresh weight and dry matter yield in connection with soil kind (sand and loamy sand); nitrogen fertiliser form (ammonium nitrate, urea, and UAN); and HA dose (0, 0.05, 0.10, and 0.15 g per kg^−^^1^ of soil).

## 3. Results

The soil type and the form of nitrogen fertilisers, as well as the organic materials in the form of HA used to aid them, had an effect on the trace element contents in the aboveground parts of maize (*Zea mays* L.) (Table 1, Table 2 and Table 3).

The differences in the trace element contents in the aboveground parts of maize between the soils under study were rather great (Table 1, Table 2 and Table 3). For most trace elements, their contents in maize were lower in the second soil (loamy sand) than in the first soil (sand) and amounted to, on average, 18% (iron), 19% (manganese), 23% (copper and cobalt), 31% (nickel), and 61% (zinc). Inverse relationships were demonstrated in the presence of chromium, lead, and cadmium. Their contents in the aboveground parts of maize were greater by 32%, 62%, and 74%, respectively, in loamy sand than in sand. As regards lead, chromium, nickel, and zinc, the greatest differences between the test soils were noted in the series with ammonium nitrate, while for cadmium, copper, and iron, in the objects with urea, and for manganese, in the pots with UAN. The variations in the contents of the most trace elements in maize cultivated in loamy sand and sand was the lowest in the objects with UAN. 

Urea fertilisation contributed to an increase in the contents of cadmium (by an average of 6% in sand and by 49% in loamy sand), lead (by 61% in sand and by 11% in loamy sand), chromium (by 61% in sand and by 11% in loamy sand), nickel (by 11% in sand and 110% in loamy sand), copper (by 10% in sand), and cobalt (by 18% in sand), as well as to a decrease in the contents of manganese (37% in loamy sand), iron (by 25% in sand and by 32% in loamy sand), zinc (by 16% in sand), and cobalt (by 44% in loamy sand), as compared to the objects with ammonium nitrate (Table 1, Table 2 and Table 3). The UAN increased, even more than urea did, the contents of chromium (by an average of 53% in sand and 26% in loamy sand), lead (by 90% in sand), manganese (by 36% in sand), zinc (by 14% in loamy sand), and cobalt (by 37% in sand), while decreasing the contents of zinc (by 24% in sand) and iron (by 48% in sand and 31% in loamy sand) in the aboveground parts of maize, as compared to ammonium nitrate. The effect of UAN on cadmium, nickel, manganese, and cobalt in plants was less unambiguous. In loamy sand, the impact of UAN was greater on the cadmium and nickel contents in maize and smaller than the effect of ammonium nitrate on the manganese and cobalt contents. In sandy, these relationships were reversed.

The impact of HA on the trace element contents in the aboveground parts of maize was indicative of dependence on the nitrogen fertiliser form and the soil type (Table 1, Table 2 and Table 3). Their effect on the contents of the elements under study was more unambiguous in the objects fertilised with ammonium nitrate than on those fertilised with urea and UAN. In the series with ammonium nitrate, the highest dose of HA (0.15 g kg^−^^1^) reduced the contents of manganese by 19%, zinc by 32%, iron by 44%, cobalt by 48%, and cadmium by 62% in sand and the contents of copper by 23%, iron by 28%, manganese by 30%, zinc by 56%, and cobalt by 63% in the aboveground parts of maize in loamy sand, as compared to the control object, i.e. that with no HA applied (Table 1, Table 2 and Table 3). It also increased the chromium content by 21% in the plants but only in sand. The first dose of HA (0.05 g · kg^−^^1^) contributed to an increase in the zinc content in sand and in the chromium and nickel contents in loamy sand, while their second dose (0.10 g kg^−^^1^) contributed to an increase in the nickel and copper contents in maize in sand. Higher doses of HA had a negative effect on their content in the plants. 

The effect of HA on the trace element contents in the plants on objects with urea applied was less unambiguously targeted than in the series with ammonium nitrate (Table 1, Table 2 and Table 3). However, their reducing impact on the chromium and nickel contents in loamy sand, the manganese and cobalt contents in sand, and the copper and iron contents in maize in both soils were noted. The first (0.05 g kg^−^^1^) or the second dose (0.10 g kg^−^^1^) of HA increased the cadmium contents in both soils; the chromium, nickel, and zinc contents in sand; and the manganese content in the plants in loamy sand. Higher doses of HA reduced the contents of these elements in maize. The highest zinc content in maize in the series with urea in loamy sand was noted following the application of the last dose of HA (0.15 g kg^−^^1^).

The application of HA in the objects with UAN also contributed to a reduction in the contents of copper by 12%, manganese by 33%, zinc by 40%, nickel by 44%, and cadmium by 52% in loamy sand, and the iron content by 63% in the aboveground parts of maize in sand (Table 1, Table 2 and Table 3). The first (0.05 g kg^−^^1^) and the second doses (0.10 g kg^−^^1^) of HA, however, increased the cadmium, nickel, zinc, and manganese contents in the plants growing in sand, and the chromium content in both soils. Higher doses of HA had a reducing effect on the contents of these elements in the plants. Under the influence of their highest dose (0.15 g kg^−^^1^), an increase was noted in the copper content in maize in sand and in the cobalt content in both soils.

**Figure 1:**
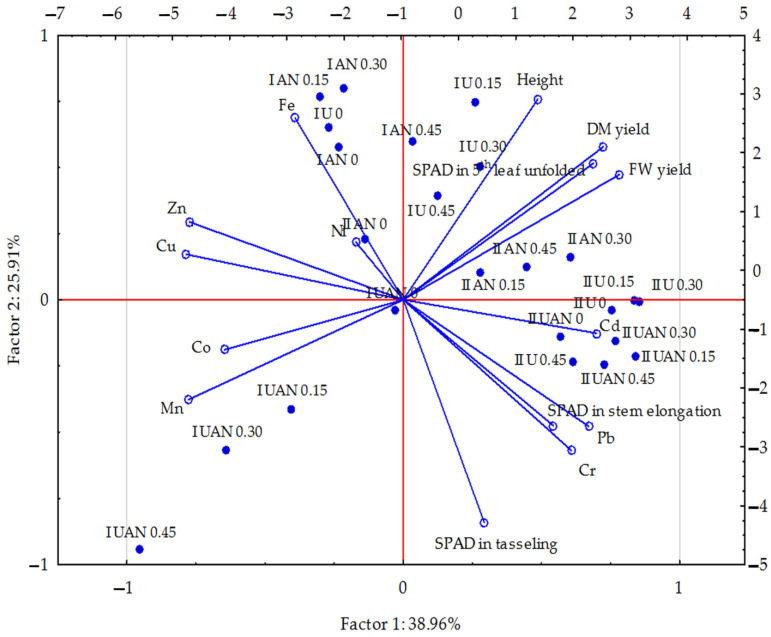
Content of trace elements in the aerial parts of maize (*Zea mays* L.) calculated with the PCA method. Key: vectors represent variables (content of Cd, Pb, Cr, Ni, Zn, Cu, Mn, Fe, and Co; leaf greenness (SPAD) index in stages of the 5th leaf unfolded, stem elongation, and tasselling; height; aerial parts fresh weight and dry matter yield; and points show the samples with elements (I—sand, II—loamy sand, AN—ammonium nitrate, U—urea, and UAN—solution of urea and ammonium nitrate using 0, 0.05, 0.10, and 0.15 g HA per kg^−1^ of soil).

A PCA analysis (Figure 1) indicated that the parameters describing the trace element content in the aboveground parts of maize were predominantly in the first group, accounting for 38.96%, and only some of them, e.g., the cadmium, copper, zinc, cobalt, or manganese contents, were in the second group (25.91% of the data set correlation). The nickel, cadmium, and cobalt content vectors were shorter than the other ones, which is indicative of their smaller effect on the factors under study. The strongest positive correlations occurred between zinc and copper and between lead and chromium, while weaker ones were noted between manganese and cobalt and between chromium and cadmium. A positive correlation was also noted between the chromium and lead contents in the plants and the SPAD index in the leaves at the stem elongation stage. Strong negative correlations were noted between cadmium and zinc, as well as between copper and cobalt, and slightly weaker correlations between lead and chromium and zinc and copper and iron. Negative correlations were also demonstrated between the manganese and cobalt contents (and weaker ones between copper and zinc) and the maize fresh and dry matter yields and between the iron, zinc, and copper contents in maize and the SPAD index in the leaves at the stem elongation stage and, also, between the manganese and cobalt contents in maize and the height and the SPAD index in the leaves at the beginning of plant growth (at the five-leaf stage). The scatter of data in Figure 1 shows that a greater effect of HA (in particular, the medium and the highest doses) on the trace element contents in maize occurred on objects with ammonium nitrate than on those with urea and UAN. The SPAD index value in the leaves at individual growth stages, the plant height, and the maize aboveground part fresh and dry matter yields were provided in a previously published paper [46].

**Figure 2:**
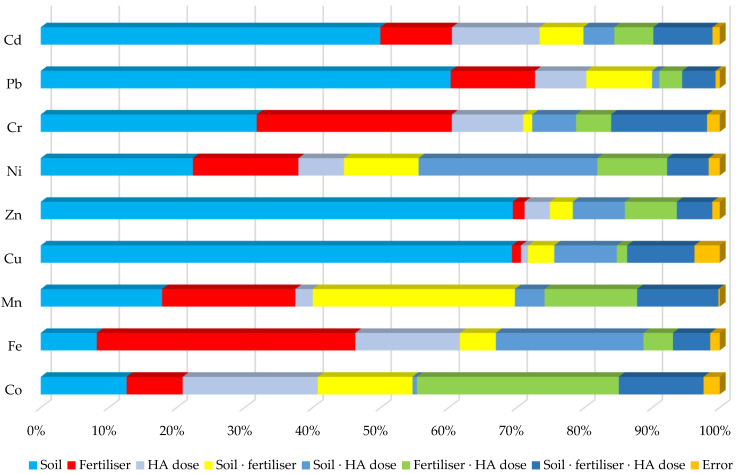
Relatively effect of factors on the contents of trace elements in maize—*Zea mays* L. (in percent). Key: contents of Cd, Pb, Cr, Ni, Zn, Cu, Mn, Fe, and Co; HA—humic acids.

Figure 2 shows the cumulative effect of the factors under study on the trace element contents in maize, expressed as a percentage of the observed variation. It follows from statistical calculations that the soil type had the greatest effect on the contents of most trace elements in the aboveground parts of maize—in particular, on zinc (69.4%), copper (69.3%), lead (60.3%), cadmium (50.0%), chromium (31.8%), nickel (22.4%), and manganese (17.9%). The nitrogen fertilisation form had the strongest effect on the contents of iron (38.0%) and manganese (19.6%) and on the contents of chromium (28.7%), nickel (15.5%), and lead (12.4%) in maize. The impact of HA on the trace element content in maize was weaker than nitrogen fertilisation and the soil type. HA had the strongest effect on the cobalt content in the plants (19.9%), as well as on the iron (15.4%) and cadmium (12.9%) contents. It should also be stressed that HA exerted the strongest effect in interactions with nitrogen fertilisers and the soil type also in interactions with these fertilisers (particularly on cobalt and manganese). 

## 4. Discussion

In the authors’ own study, the application of nitrogen fertilisers had a significant effect on an increase in the contents of all trace elements of the aboveground parts of maize. The greatest differences in the trace element contents in the biomass of the test plant cultivated in loamy sand and sand were noted in the series with ammonium nitrate, while the smallest differences were in the objects with UAN. Similar results were obtained by Wyszkowski and Brodowska [59], who noted an increase in the contents of manganese by 26% and iron by 66% in the aboveground parts of maize and elevated Fe:Zn and Fe:Mn ratios under the influence of fertilisation with an urea–ammonium nitrate solution (N—280 g kg^−^^1^). Under the same conditions, the contents of cadmium by 26%, nickel by 27%, lead by 37%, and cobalt by 47% in the plant decreased. As reported by the authors, nitrogen fertilisation caused ambiguous changes in the trace element bioconcentration factors in the aboveground parts of maize, which is also consistent with the results of the presented study. Zhou [60] noted the stimulating effect of nitrogen fertilisation (in the form of NH_4_NO_3_) on the cadmium and lead contents in radishes, carrots, and potatoes. Razanov et al. [61] also demonstrated that the use of mineral fertilisation, particularly ammonium nitrate (60 kg ha^−^^1^), promoted the accumulation of lead, cadmium, and zinc in the leaves and seeds of *Silybum marianum* L. These authors also noted high rates of accumulation of the analysed elements, which was indicative of their intensive uptake by plants at concentrations much higher than the contents of their mobile forms in the soil. The effect of nitrogen fertilisation on the trace element contents in plants results mainly from the yield-forming action of nitrogen, its effect on the biological value of the yield, and the change in soil pH in the root zone of plants [62]. By reducing the pH value of the soil, mineral nitrogen fertilisers increase the mobility and bioavailability of trace elements in the soil–plant system [63]. The long-term use of mineral fertilisers can considerably affect trace element accumulation in the soil [64]. In an experiment conducted by Wyszkowski and Brodowska [65], nitrogen fertilisation (UAN at a dose of 130 and 170 mg N kg soil^−^^1^) increased the contents of zinc by 3%, iron by 3%, copper by 9%, manganese by 12%, chromium by 15%, and cobalt by 59% in the soil. Analogous observations and conclusions were also presented by Singh et al. [66], as well as Zhao et al. [67]. Fertilisation can significantly modify the soil environment through the effect on the pH, organic substance content, sorption capacity, and granulometric composition and thus affect the trace element phytoavailability [68].

The results obtained in this study showed a positive (reducing) effect of HA on the trace element contents in the aboveground maize biomass. Their strongest effect was noted in the series with ammonium nitrate. A similar tendency was noted by Rong et al. [48], who assessed the effect of HA (6.4, 10.3, and 14.8 kg ha^−1^) on the contents of available cadmium, copper, lead, and zinc fractions in the soil and the accumulation of these elements in tobacco leaves. These authors demonstrated that the application of HA at the highest dose reduced the contents of lead, cadmium, zinc, and copper in the soil by 39%, 37%, 29%, and 18%, respectively, as compared to the control series. The incorporation of HA into the soil also contributed to a reduction in the accumulation of the analysed elements in tobacco leaves. The researchers believed that the results obtained confirmed the trace element binding capacity by the functional groups of HA [69], which fundamentally changes the mobility of these elements in the soil and, thus, their phytoavailability. A positive correlation between the application of HA and FA (4 g kg^−1^) and the cadmium and lead contents in tobacco leaves was also demonstrated by Zhang et al. [70]. However, as demonstrated by the authors, the stimulating effect of humic substances on trace element availability is only achievable in acidic soils. The lead leaf content was decreased by 18-48%, with the cadmium concentration by 9–33% after the application of 4 g kg^−1^ HA and FA to the acidic soils (in reverse to alkaline soils). In an experiment conducted by Yildirim et al. [71], the addition of HA+FA (7000 mg L^−1^) reduced the cadmium and chlorine contents in the aboveground parts of *Lepidium sativum* cultivated in a soil contaminated with cadmium (10 mg kg^−1^) and lead (500 mg kg^−1^) (by 95% and 80%, respectively), as compared to the control series. The important role of humic substances in shaping the processes of trace element immobilisation, toxicity, and availability was also demonstrated by Yang et al. [72].

According to Zhou et al. [73], the bioavailability and mobility of zinc and lead in the soil environment can be reduced (by 33–34%) by maintaining an appropriate soil pH value and introducing humic substances that increase the sorption capacity of soils. As a consequence of these activities, the mobility of trace elements is reduced due to the complexation, precipitation of insoluble phases, and adsorption by HA. Certain scientific experiments indicate that humic substances can affect the pH value of the soil [74], as well as the exchange capacity [75]. The soil organic substances include a number of active functional groups (e.g., –OH, –COOH, –NH_3_, and –OCH_3_), which can form complexes with the trace element cations, which are known as chelates [76,77]. The formation of these organometallic connections in the soil is of extreme importance, as it prevents the leaching of trace element ions from the soil, partially detoxicates them, and reduces their uptake by plants [76]. Therefore, the constant attention to replenishing soils with organic matter is important. In this case, it is preferable to use humic substances rather than straw or other materials subject to rapid mineralisation. The use of straw for fertilisation can incorporate into the soil low-molecular-weight organic matter, which acts as a trace element carrier [78]. The products of straw mineralisation, including organic acids, increase the amount of their phytoavailable forms in the soil through the formation of small-molecule connections with elements [79]. 

Not only does the use of mineral fertilisers provide plants with nutrients, but it also changes the availability of trace elements in the soil and their absorption by plants. For this reason, research focused on the search for alternative solutions in plant fertilisation (e.g., biostimulants in the form of humic substances), which can contribute to improved production efficiency while being environmentally safe, remains relevant and topical. 

## 5. Conclusions

The plants cultivated in loamy sand contained less iron, manganese, copper, cobalt, nickel, and zinc while containing more chromium, lead, and cadmium than in sand. Urea fertilisation increased the cadmium, lead, chromium, and nickel contents and reduced the iron content in maize in both soils. UAN contributed to an increased chromium content, being higher than that caused by urea, and to a reduced iron content in the aboveground parts of maize, as compared to the objects with ammonium nitrate. The direction of changes in the contents of other trace elements under the influence of urea and UAN varied and was dependent on the soil type. The effect of HA on the trace element contents in the aboveground parts of maize was dependent on the nitrogen fertiliser form and the type of soil. In the series with ammonium nitrate, the highest dose of HA reduced the manganese (19–30%), zinc (32–56%), iron (28–44%), and cobalt (48–63%) contents in maize in both soils, as compared to the control object (with no HA). The impact of HA on the trace element contents in plants in the objects fertilised with urea and UAN was less clearly targeted than that in the series with ammonium nitrate. In the series with urea, however, their reducing effect on the copper (4–5%) and iron (42–63%) contents in maize in both soils was noted. The study also demonstrated a positive reduction in the contents of many other trace elements in maize under the influence of the application of HA (particularly, their highest dose). However, it only concerned one of the soils under study. As regards the second soil, no effect was noted or reverse relationships were found. The application of HA into the soil can be effective in reducing the trace element contents in plants and can mitigate the adverse environmental impact of intensive agricultural production.

## Figures and Tables

**Table 1:** Content of cadmium, lead, and chromium in maize—*Zea mays* L. (mg kg^−1^ DM).

HA Dose g kg^−1^ of Soil	Sand				Loamy Sand			
	Ammonium Nitrate	Urea	UAN	Average	Ammonium Nitrate	Urea	UAN	Average
Cadmium content (mg kg^−1^ DM)								
0	0.135 ^g^	0.071 ^a−c^	0.068 ^ab^	0.091 ^A^	0.123 ^e–g^	0.180 ^h^	0.175 ^h^	0.159 ^D^
0.05	0.101 ^de^	0.106 ^d–f^	0.073 ^a–c^	0.093 ^A^	0.174 ^e–g^	0.239 ^i^	0.185 ^h^	0.199 ^F^
0.10	0.093 ^cd^	0.138 ^g^	0.119 ^e–g^	0.117 ^B^	0.128 ^fg^	0.222 ^i^	0.176 ^h^	0.175 ^E^
0.15	0.051 ^a^	0.089 ^h^	0.093 ^b–d^	0.078 ^C^	0.123 ^e–g^	0.176 ^h^	0.084 ^b–d^	0.128 ^B^
Average	0.095 ^AB^	0.101 ^B^	0.088 ^A^	0.095 ^A^	0.137 ^C^	0.204 ^E^	0.155 ^D^	0.165 ^B^
*r*	−0.972	0.389	0.674	−0.141	−0.240	−0.120	−0.766	−0.511
Lead content (mg kg^−1^ DM)								
0	0.462 ^b^	0.610 ^c^	0.739 ^d–f^	0.604 ^A^	0.934 ^ij^	1.036 ^kl^	0.841 ^gh^	0.937 ^E^
0.05	0.255 ^a^	0.677 ^c–e^	0.754 ^ef^	0.562 ^D^	0.770 ^f−g^	0.893 ^h–j^	0.883 ^hi^	0.849 ^B^
0.10	0.321 ^a^	0.461 ^b^	0.671 ^cd^	0.484 ^C^	0.908 ^h–j^	0.969 ^jk^	0.688 ^de^	0.855 ^B^
0.15	0.472 ^b^	0.689 ^de^	0.715 ^d–f^	0.625 ^A^	0.934 ^i–j^	1.049 ^l^	1.135 ^m^	1.039 ^F^
Average	0.378 ^B^	0.609 ^C^	0.720 ^D^	0.569 ^A^	0.887 ^A^	0.987 ^E^	0.887 ^A^	0.920 ^B^
*r*	0.116	0.026	−0.552	−0.026	0.227	0.207	0.478	0.454
Chromium content (mg ^kg−1^ DM)								
0	1.132 ^ab^	0.947 ^a^	1.757 ^e–g^	1.279 ^F^	1.486 ^cd^	2.355 ^ij^	2.148 ^h−j^	1.996 ^DE^
0.05	1.330 ^bc^	1.193 ^ab^	1.987 ^gh^	1.503 ^A^	2.134 ^h–j^	2.118 ^hi^	2.369 ^h–j^	2.207 ^F^
0.10	1.204 ^ab^	2.047 ^h^	2.384 ^j^	1.878 ^BC^	1.925 ^f–h^	2.005 ^gh^	2.399 ^j^	2.110 ^EF^
0.15	1.371 ^bc^	1.659 ^d–e^	1.563 ^c–e^	1.531 ^A^	1.665 ^d–f^	1.685 ^d–f^	2.178 ^h−j^	1.843 ^B^
Average	1.259 ^A^	1.462 ^B^	1.923 ^D^	1.548 ^A^	1.803 ^C^	2.041 ^E^	2.274 ^F^	2.039 ^B^
*r*	0.689	0.789	−0.068	0.590	0.148	−0.984	0.120	−0.460

HA—humic acids, r—correlation coefficient. Values with different letters (^A–F^ for HA dose, nitrogen fertiliser form, and the kind of soil, ^a–m^ for interaction between HA dose, nitrogen fertiliser form, and the kind of soil) are significantly different at *p* ≤ 0.01 (SNOVA and Tukey’s HSD test).

**Table 2:** Content of nickel, zinc and copper in maize—*Zea mays* L. (mg kg^−1^ DM).

HA Dose g kg^−1^ of Soil	Sand				Loamy Sand			
	Ammonium Nitrate	Urea	UAN	Average	Ammonium Nitrate	Urea	UAN	Average
Nickel content (mg kg^−1^ DM)								
0	1.258 ^cd^	2.194 ^fg^	1.315 ^cd^	1.589 ^AB^	0.432 ^a^	2.160 ^fg^	2.160 ^fg^	1.584 ^AB^
0.05	1.348 ^cd^	2.352 ^hi^	1.414 ^d^	1.705 ^BC^	1.526 ^de^	2.103 ^fg^	1.830 ^ef^	1.820 ^C^
0.10	3.216 ^j^	2.716 ^i^	2.357 ^hi^	2.763 ^D^	0.951 ^bc^	2.037 ^fg^	1.217 ^b–d^	1.402 ^A^
0.15	3.145 ^j^	2.717 ^i^	2.060 ^fg^	2.641 ^D^	0.834 ^ab^	1.554 ^de^	1.219 ^b–d^	1.202 ^E^
Average	2.242 ^E^	2.495 ^F^	1.787 ^C^	2.174 ^B^	0.936 ^A^	1.964 ^D^	1.607 ^B^	1.502 ^A^
*r*	0.896	0.945	0.814	0.887	0.180	−0.876	−0.947	−0.767
Zinc content (mg kg^−1^ DM)								
0	15.08 ^h−j^	12.94 ^gh^	10.07 ^ef^	12.70 ^E^	8.93 ^de^	3.74 ^ab^	7.58 ^cd^	6.75 ^C^
0.05	22.24 ^k^	13.36 ^g−i^	11.74 ^fg^	15.78 ^B^	4.22 ^ab^	3.35 ^bc^	5.79 ^bc^	4.45 ^A^
0.10	16.89 ^j^	15.47 ^ij^	17.13 ^j^	16.50 ^B^	3.89 ^ab^	5.94 ^fg^	6.03 ^bc^	5.29 ^A^
0.15	10.20 ^ef^	12.18 ^fg^	10.26 ^ef^	10.88 ^D^	3.89 ^ab^	8.15 ^c−e^	4.53 ^ab^	5.52 ^A^
Average	16.10 ^D^	13.49 ^C^	12.30 ^B^	13.96 ^B^	5.23 ^A^	5.30 ^A^	5.98 ^A^	5.50 ^A^
*r*	−0.519	−0.016	0.233	−0.232	−0.808	0.921	−0.919	−0.387
Copper content (mg kg^−1^ DM)								
0	1.771 ^ab^	2.408 ^gh^	2.075 ^d–f^	2.085 ^D^	2.036 ^c–e^	1.698 ^ab^	1.781 ^a−c^	1.838 ^C^
0.05	2.186 ^e–h^	2.378 ^gh^	2.126 ^d–g^	2.230 ^A^	1.916 ^b–d^	1.792 ^a–c^	1.728 ^ab^	1.812 ^C^
0.10	2.370 ^gh^	2.336 ^gh^	2.224 ^e–h^	2.310 ^A^	1.544 ^a^	1.660 ^ab^	1.767 ^ab^	1.657 ^B^
0.15	2.212 ^e–h^	2.297 ^f–h^	2.357 ^gh^	2.289 ^A^	1.561 ^a^	1.630 ^a^	1.561 ^a^	1.584 ^B^
Average	2.135 ^B^	2.355 ^C^	2.196 ^B^	2.228 ^B^	1.765 ^A^	1.695 ^A^	1.709 ^A^	1.723 ^A^
*r*	0.761	−0.998	0.982	0.880	−0.930	−0.616	−0.791	−0.969

*r*—correlation coefficient. Values with different letters (^A–F^ for HA dose, nitrogen fertiliser form, and the kind of soil, and ^a–k^ for interactions between the HA dose, nitrogen fertiliser form, and the kind of soil) are significantly different at *p* ≤ 0.01 (ANOVA and Tukey’s HSD test).

**Table 3:** Content of manganese, iron, and cobalt in maize—*Zea mays* L. (mg kg^−1^ DM).

HA Dose g kg^−1^ of Soil	Sand				Loamy Sand			
	Ammonium Nitrate	Urea	UAN	Average	Ammonium Nitrate	Urea	UAN	Average
Manganese content (mg kg^−1^ DM)								
0	74.14 ^j^	73.95 ^j^	80.84 ^k^	76.31 ^B^	102.59 ^m^	44.22 ^a^	67.50 ^h^	71.44 ^A^
0.05	69.32 ^hi^	63.95 ^fg^	84.16 ^kl^	72.48 ^AB^	85.08 ^l^	46.67 ^a^	53.94 ^b^	61.90 ^E^
0.10	74.05 ^j^	62.50 ^ef^	110.63 ^n^	82.39 ^F^	57.66 ^cd^	55.93 ^bc^	66.51 ^gh^	60.03 ^D^
0.15	59.99 ^de^	72.01 ^ij^	101.69 ^m^	77.90 ^B^	72.16	53.55 ^b^	45.08 ^a^	56.93 ^C^
Average	69.38 ^A^	68.10 ^A^	94.33 ^E^	77.27 ^B^	79.37 ^D^	50.09 ^B^	58.26 ^C^	62.57 ^A^
*r*	−0.732	−0.164	0.809	0.462	−0.802	0.867	−0.658	−0.937
Iron content (mg kg^−1^ DM)								
0	39.52 ^i^	32.59 ^h^	22.03 ^e–g^	31.38 ^D^	35.14 ^h^	20.39 ^d–f^	16.98 ^cd^	24.17 ^A^
0.05	35.93 ^hi^	32.08 ^h^	20.06 ^d–f^	29.36 ^D^	20.39 ^a^	11.92 ^ab^	12.98 ^bc^	15.10 ^B^
0.10	35.26 ^h^	18.47 ^de^	19.29 ^d–f^	24.34 ^A^	23.11 ^fg^	19.74 ^d–f^	18.79 ^de^	20.55 ^C^
0.15	22.00 ^e–g^	16.63 ^cd^	8.08 ^a^	15.57 ^B^	25.32 ^g^	18.28 ^de^	23.18 ^fg^	22.26 ^AC^
Average	33.18 ^C^	24.94 ^B^	17.37 ^A^	25.16 ^B^	25.99 ^B^	17.58 ^A^	17.98 ^A^	20.52 ^A^
*r*	−0.894	−0.926	−0.874	−0.961	−0.537	0.050	0.745	−0.009
Cobalt content (mg kg^−1^ DM)								
0	0.487 ^g^	0.694 ^h^	0.466 ^fg^	0.549 ^F^	0.652 ^h^	0.249 ^a–d^	0.320 ^b–e^	0.407 ^BC^
0.05	0.463 ^fg^	0.378 ^ef^	0.529 ^g^	0.457 ^D^	0.552 ^g^	0.223 ^ab^	0.337 ^de^	0.371 ^AB^
0.10	0.296 ^a–e^	0.226 ^ab^	0.510 ^g^	0.344 ^A^	0.236 ^a–c^	0.205 ^a^	0.331 ^c–e^	0.257 ^E^
0.15	0.252 ^a–d^	0.464 ^fg^	0.549 ^g^	0.422 ^CD^	0.243 ^a–d^	0.266 ^a–d^	0.469 ^fg^	0.326 ^A^
Average	0.375 ^A^	0.441 ^B^	0.514 ^D^	0.443 ^B^	0.421 ^B^	0.236 ^C^	0.364 ^A^	0.340 ^A^
*r*	−0.955	−0.556	0.838	−0.751	−0.934	0.157	0.811	−0.714

*r*—correlation coefficient. Values with different letters (^A–F^ for HA dose, nitrogen fertiliser form, and the kind of soil, ^a–n^ for interactions between HA dose, nitrogen fertiliser form, and the kind of soil) are significantly different at *p* ≤ 0.01 (ANOVA and Tukey’s HSD test).

## Data Availability

Data are available by contacting the authors.
